# Global trends in clinical trials and interventions for the metabolic syndrome: A comprehensive analysis of the WHO International Clinical Trials platform

**DOI:** 10.1016/j.conctc.2024.101330

**Published:** 2024-06-22

**Authors:** Ndivhuwo Muvhulawa, Phiwayinkosi V. Dludla, Musawenkosi Ndlovu, Yonela Ntamo, Asanda Mayeye, Nomahlubi Luphondo, Nokulunga Hlengwa, Albertus K. Basson, Sihle E. Mabhida, Sidney Hanser, Sithandiwe E. Mazibuko-Mbeje, Bongani B. Nkambule, Duduzile Ndwandwe

**Affiliations:** a Cochrane South Africa, South African Medical Research Council, Tygerberg, 7505, South Africa; b Department of Biochemistry, North-West University, Mafikeng Campus, Mmabatho, 2735, South Africa; c Department of Biochemistry and Microbiology, University of Zululand, KwaDlangezwa, 3886, South Africa; d Non-Communicable Diseases Research Unit, South African Medical Research Council, Tygerberg, 7505, South Africa; e Department of Physiology and Environmental Health, University of Limpopo, Sovenga, 0727, South Africa; f School of Laboratory Medicine and Medical Sciences, University of KwaZulu-Natal, Durban, 4000, South Africa

**Keywords:** Metabolic syndrome, Global trends, International Clinical Trials Registry Platform (ICTRP), Demographic characteristics, Treatment modalities

## Abstract

Metabolic syndrome has emerged as a significant global public health concern, necessitating comprehensive examination alongside cardiovascular diseases (CVDs) and type 2 diabetes mellitus (T2D). This study provides a comprehensive analysis of clinical trials, drawing upon data sourced from the International Clinical Trials Registry Platform (ICTRP), until April 2023. Information pertaining to trial attributes and intervention features was gathered and subsequently summarized. Among the 2379 studies found on ICTRP from 18 clinical registries, ClinicalTrials.gov was the most popular with 55 % of the studies, based on data emerging from the United States. Most trials were for treatment (44 %) and prevention (17 %), with fewer focused on basic science, and diagnostic purposes. Diet and exercise were the most prominent, with 710 and 247 studies, respectively. Metformin and statins emerge as leading pharmacological therapies, reflecting the prevalence of CVD and T2D in the context of metabolic syndrome. However, there is growing recognition of other promising interventions, such as Glucagon-Like Peptide-1 agonists and Dipeptidyl Peptidase IV inhibitors, which offer potential in slowing the progression of metabolic syndrome-related conditions. Notably, clinical trials primarily assessed diagnostic markers like lipid profiles, insulin, and blood pressure, rather than body mass and body mass index. These parameters are crucial for evaluating the effectiveness and safety of interventions for metabolic syndrome due to its multi-condition nature. Most studies aimed to address general symptom relief, while highlighting a need for additional well-designed treatment trials with rigorous methodologies in accordance with the World Health Organization's guidance for consistent evaluation and treatment.

## 1 Introduction

Metabolic syndrome refers to a cluster of cardiometabolic abnormalities that are consistent with the development of cardiovascular disease (CVD) and type 2 diabetes (T2D) [1,2]. Overnutrition, concomitant with undesirable lifestyle modification contributes to the initiation of obesity [3], which is the major contributor to the development of metabolic syndrome [4]. The global prevalence of obesity has reached alarming levels, which is estimated at 14 % of the global adult population, and steadily increasing [5]. In recent times, the topic of metabolic syndrome has received great attention as the coexistence of the diseases falling under the umbrella are no longer known to occur in developed countries [6], but now becoming more prominent within developing nations [7,8]. This health issue surpasses socioeconomic and ethnic boundaries and serves as a precursor to metabolic syndrome, a collection of risk factors such as central obesity, insulin resistance, dyslipidemia, and hypertension [4,9,10].

Therefore, it remains imperative to continually monitor the global trends in disease growth, while putting in place effective interventions to curb the rapid prevalence of metabolic syndrome at global stage. Numerous studies have reported that the metabolic syndrome, not only increases the risk of CVD and T2D but is also linked to the development of several comorbidities including non-alcoholic steatohepatitis, reproductive disorders, pro-inflammatory diseases, as well as certain types of cancers [4,9,10]. The complicated interplay between obesity, metabolic syndrome, and their association with these diseases highlights the urgent need for comprehensive public health strategies aimed at preventing and managing this prevalence.

Since its inception in 2006, the International Clinical Trials Registry Platform (ICTRP) has become important for the advancement of knowledge regarding the global trends in disease prevalence [11]. By serving as a comprehensive repository for global clinical trial data, ICTRP enables researchers and healthcare professionals to retrieve valuable information on metabolic disease studies. In fact, increasing studies have explored some of the strengths and weaknesses of using this registry to report on data integrity and the trends in clinical trials, diseases, and drugs [12,13]. However, due to the increasing number of studies making use of this platform [[14], [15], [16]], the ICTRP remains relevant in facilitating the systematic analysis of clinical trials related to metabolic diseases, such as diabetes and obesity, on an international scale. As such, this study remains important to critically analyse clinical trials registered within this portal to compare data from diverse world regions and populations, helping identify emerging trends, treatment effectiveness, and potential variations in disease management strategies. This is also relevant for development of evidence-based interventions and strategies to combat the rising prevalence of metabolic diseases.

## 2 Methodology

### 2.1 Source and data description

The ICTRP registry (https://www.who.int/clinical-trials-registry-platform) was used to collect data on clinical trials, offering comprehensive insights into the global trends related to metabolic syndrome across different countries worldwide [17]. This database contains clinical data from registries across the globe and has become a valuable tool to monitor disease surveillance or to evaluate the effects on health outcomes across different settings and world populations [18,19]. To identify relevant clinical trials, an advanced search was conducted on the ICTRP using the keyword “metabolic syndrome” and other related terms like “metabolic disease”. This search included trials registered from inception up until 18 April 2023, at 08:55 a.m. A method describing a similar search has already been described [20]. The criteria used to detect duplicated trials were as follows: trials sharing the same trial ID and those with similar public titles were considered duplicates. Additionally, matching intervention details, outcome measures, and study locations were considered.

### 2.2 An approach for data analysis and management

Two reviewers (N.M. and M.N.) individually accessed the WHO-ICTRP portal to download relevant data in April 2023. The downloaded Excel spreadsheet was checked by other researchers (P.V.D. and D.N.) before data extractions was initiated, while collecting data concerning trial registry source, date of registration, gender, and intervention model. Other relevant information that was collected was based on disease condition, primary outcomes and therapeutic interventions, corresponding to the potential management of metabolic syndrome. This information allowed for a comprehensive analysis of global trends in metabolic syndrome within an Excel spreadsheet.

## 3 Results

### 3.1 Characteristic features of included clinical trials

**Fig. 1:**
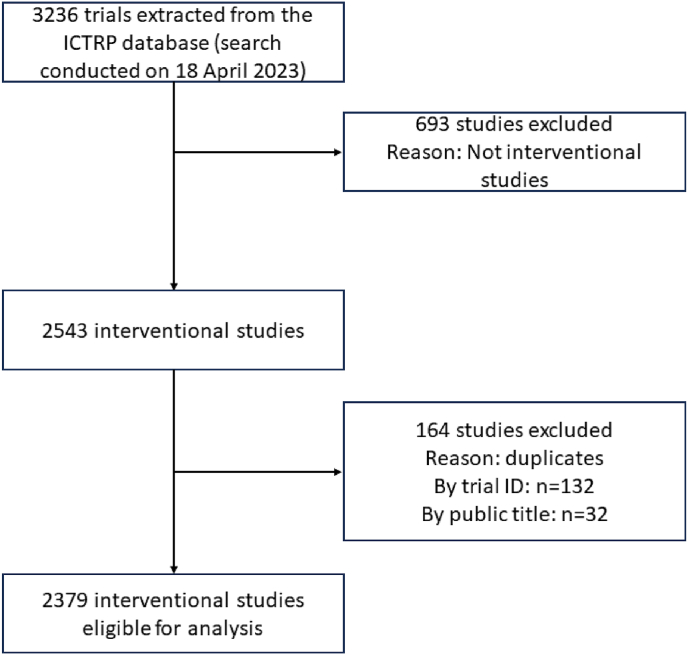
Flowchart illustrating the process of trial selection process of interventional studies for analysis from a pool of 3236 trials extracted from the International Clinical Trials Registry Platform (ICTRP) database.

**Fig. 2:**
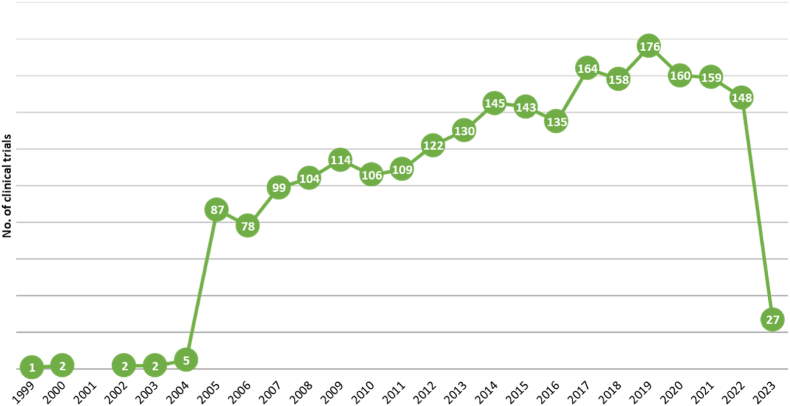
The number of trials registered, including yearly trends, with the International Clinical Trials Registry Platform (ICTRP) portal till until April 2023.

The dataset used for this analysis was downloaded in April 2023. Initially, the dataset included 3236 studies. However, certain criteria were applied to remove studies from the analysis (Fig. 1). A total of 164 duplicate studies were removed, 132 records were eliminated due to sharing the same trial ID, while 32 of these were excluded for having identical titles. The current study focused on interventional studies; thus 693 records were also removed as they did not meet the classification of interventional studies. Overall, 857 studies were eliminated, leaving a total of 2379 studies for further analysis (Fig. 1). These 2379 studies have been registered since 1999, with a rising trend until 2023 (Fig. 2). During the years 1999–2004, there were very few studies registered, the lowest being in 2001 with almost no registered clinical trials. Interestingly, there was a jump in the number of clinical trials from 2004 to 2005, which could be attributed to rising external factors like obesity [21] significantly contributing to this rapid shift, as seen in other similar studies [22]. Notably, the most significant changes occurred after 2004, where the highest reported studies were seen between 2016 and 2023, corresponding to the rising prevalence of the metabolic syndrome over the years [23]. Interestingly, the highest report was in 2019, having 176 (7.4 %) registered studies (Fig. 2).

### 3.2 Trends in global distribution of clinical trials

**Fig. 3:**
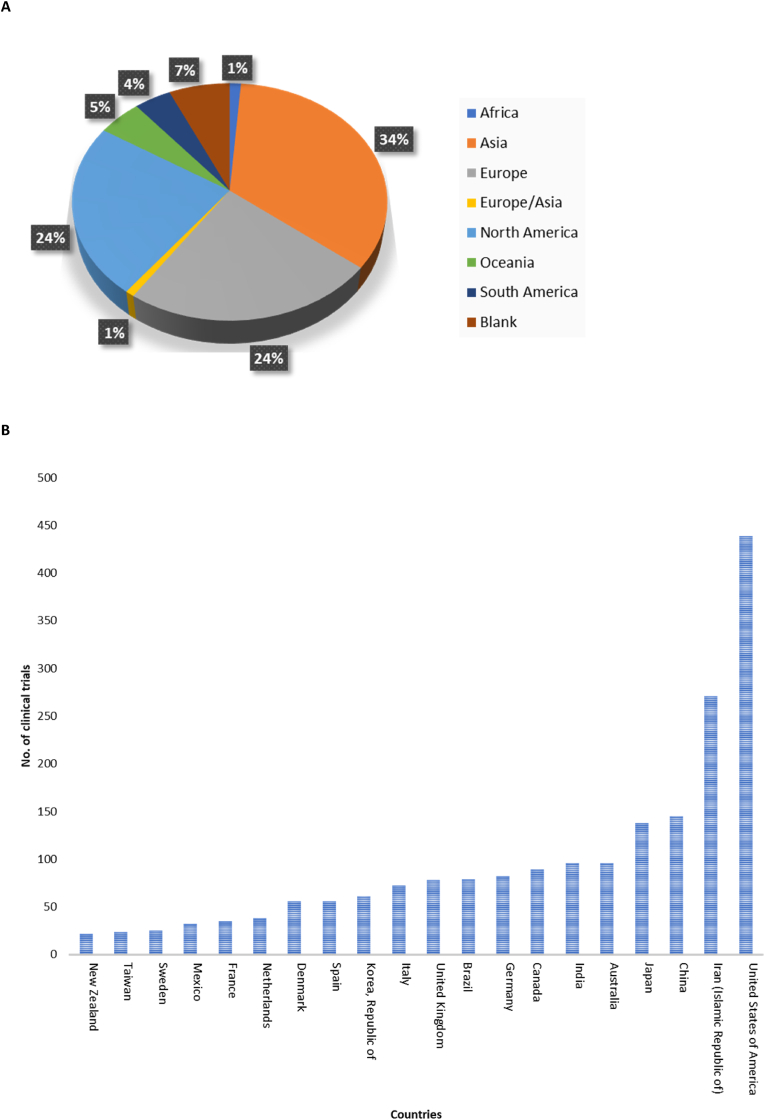

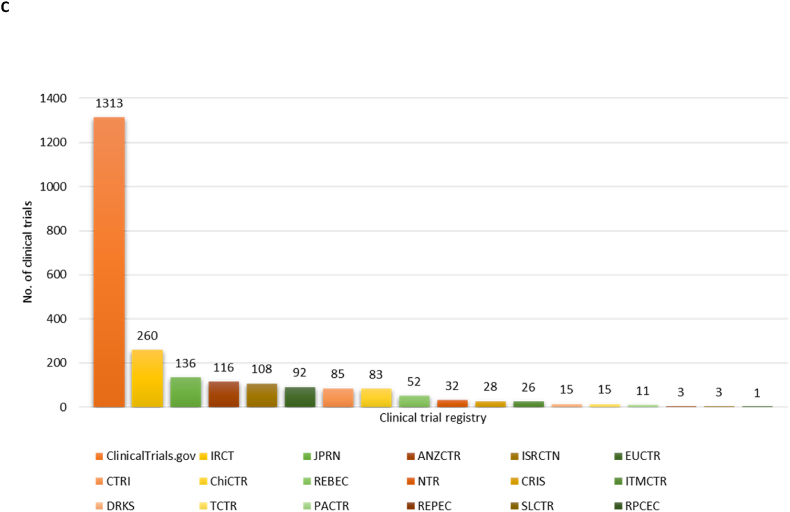
This figure provides a comprehensive overview of the global distribution of clinical trials, depicting three key aspects: (A) a pie chart representing the percentage distribution of clinical trials conducted on each continent, (B) a bar graph showing the number of trials conducted per country, and (C) a bar graph illustrating the number of studies registered across various clinical trial registries.

An analysis of registered clinical trials reveals significant activity in several continents/countries. Fig. 3A displays the percentage distribution of trials registered in each continent identified from the ICTRP database. The majority of trials originated from Asia (34 %), followed by Europe (24 %) and North America (24 %). Oceania accounted for 5 % of the trials, South America for 4 %, and Africa for 1 %. Another 1 % of the trials were from countries that span both Europe and Asia. Additionally, 7 % of the trials did not specify the study location indicated as “blank”. Fig. 3B shows some of the countries with registered clinical trials. The United States is ranked first among the 81 countries that had registered clinical trials in the ICTRP with a total of 439 studies. Asian countries show some of the highest number of registered trials, with Iran having the second highest number of 271 studies, followed by China with 145 studies and Japan with 138 studies. Interestingly, some of the countries with the lowest number of registered trials are from Asia, including Bangladesh, Iraq, Lebanon, and Saudi Arabia (not shown in Fig. 3B), all with only 1 registered clinical trial. These findings highlight the varying degrees of research engagement on metabolic syndrome in different countries.

**Fig. 4:**
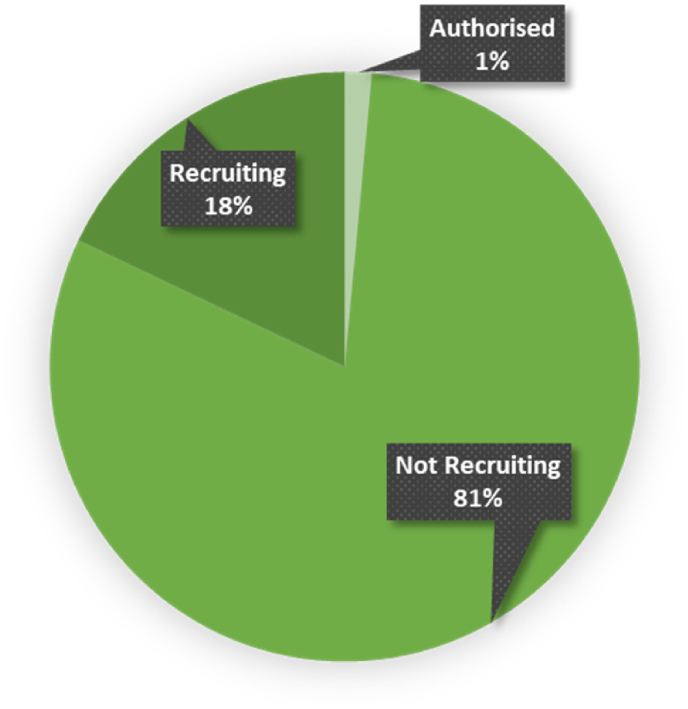
The diagram gives an overview of the recruitment status, to indicates the current stage of a trial, whether it is authorized (1 %), recruiting (18 %), or not recruiting (81 %).

The 2379 studies found on ICTRP were from 18 clinical registries. Fig. 3C represents the relative distribution of studies across these registries, providing insights into their respective popularity and utilization within the research community. Most studies were from the ClinicalTrials.gov registry (1313, 55.19 %), 260 (10.93 %) from the Iranian Registry of Clinical Trial (IRCT), 136 (5.72 %) from the Japan Primary Registries Network (JPRN), 116 (4.88 %) from the Australian New Zealand Clinical Trials Registry (ANZCTR), and 108 (4.54 %) from the International Standard Randomized Controlled Trial Number (ISRCTN) (Fig. 3C). The lowest number of studies were registered in the Pan African Clinical Trial Registry (PACTR) with only 11 (0.46 %) studies, followed by the Peruvian Clinical Trials Registry (REPEC) and the Sri Lanka Clinical Trials Registry (SLCTR) with 3 (0.13 %) studies (Fig. 3C). The lowest was also noted for the Cuban Public Registry of Clinical Trials (RPCEC), having only 1 (0.04 %) registered trial. Overall, 1913 (80.41 %) of the registered trials had a “not recruiting” status, indicating that these trials were no longer enrolling participants. In contrast, only 426 (17.97 %) studies were recruiting. Additionally, 35 trials (1.47 %) were authorized, indicating that they received approval to proceed but have not yet started recruiting participants. Lastly, 5 trials (0.21 %) did not specify their current recruitment status (Fig. 4).

### 3.3 Trends in gender and age distribution of participants included in clinical trials

**Fig. 5:**
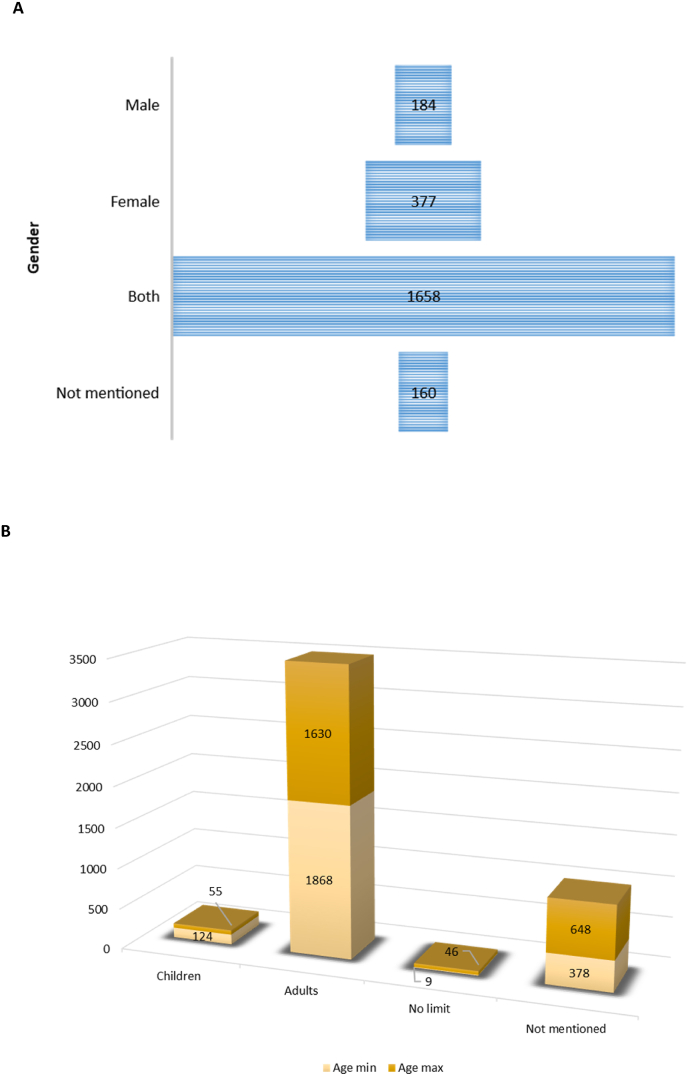
The number registered clinical trials based on gender **(A)**, while also indicating the minimum and maximum age for inclusion in clinical trials **(B),** Children = 0–17 years, adults = 18 and above.

Metabolic syndrome clinical trials investigations predominantly included both male and female participants (1658, 69.69 %), whereas 377 (15.85 %) of the studies focused only on females, and 184 (7.73 %) were on male participants (Fig. 5A). However, 160 (6.73 %) of studies did not mention the inclusion gender (Fig. 5A). Furthermore, 1868 studies were recruiting only adults (18 and above) whereas 124 studies had an included children as young as newborns (0–17 years) in their investigations and 378 studies did not specify the minimum inclusion age while 648 studies did not specify the maximum inclusion age (Fig. 5B). When assessing the maximum age requirement, it was evident that only 55 studies focused only on children (Fig. 5B).

### 3.4 Trends in type of clinical trials and intervention models used

**Fig. 6:**
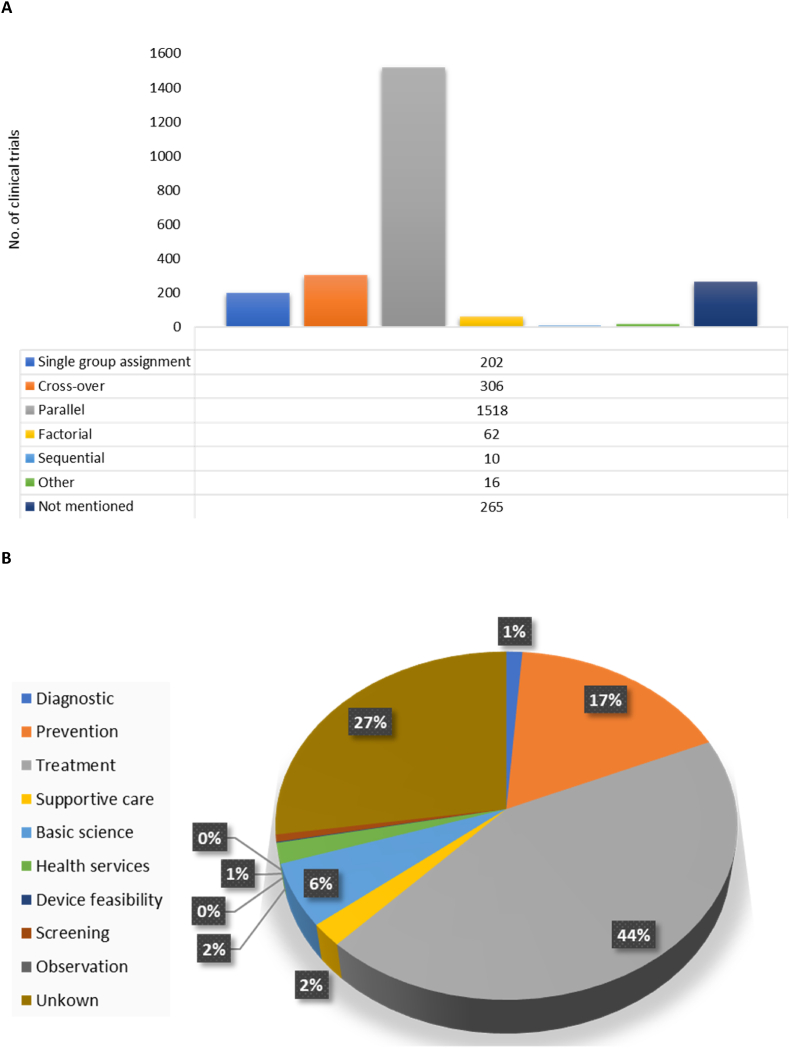
The type of intervention models used in clinical trials **(A)**, with parallel assignments being the most dominant. **(B)** Represents the different interventions used in these clinical trials. With treatment (44 %), prevention (17 %), basic science (6 %), and supportive care (2 %) being the leading primary purposes for clinical trial studies. While almost 27 % of clinical trials were unknown or uncategorized.

Further analysis of the data looking at the types of intervention models revealed that the number of clinical trials being conducted were classified as parallel assignments, having 1518 (63.81 %) registered trials, while 306 (12.86 %) were cross over, 202 (8.49 %) single-group, and the lowest number was 62 (2.61 %) for those considered to be factorial and 10 (0.42 %) sequential (Fig. 6A). Of the total studies, 265 (11.14 %) were not specified and 16 (0.67 %) were categorized as other (Fig. 6A). Many of the studies were for treatment (1037, 43.59 %) and prevention (409, 17.19 %) purposes (Fig. 6B). Fewer studies were registered for basic science (140, 5.88 %), supportive care (51, 2.14 %), health services (45, 1.89 %), and diagnostic (29, 1.22 %) purposes (Fig. 6B). Less than 1 % of studies are focused on screening (16, 0.67), device feasibility (2, 0.08 %), and observation (0, 0.00 %). However, a great portion (650, 27.32 %) of the study's primary purposes were unknown (Fig. 6B).

### 3.5 Trends in therapeutic interventions, disease conditions and primary outcomes of clinical trials

**Fig. 7:**
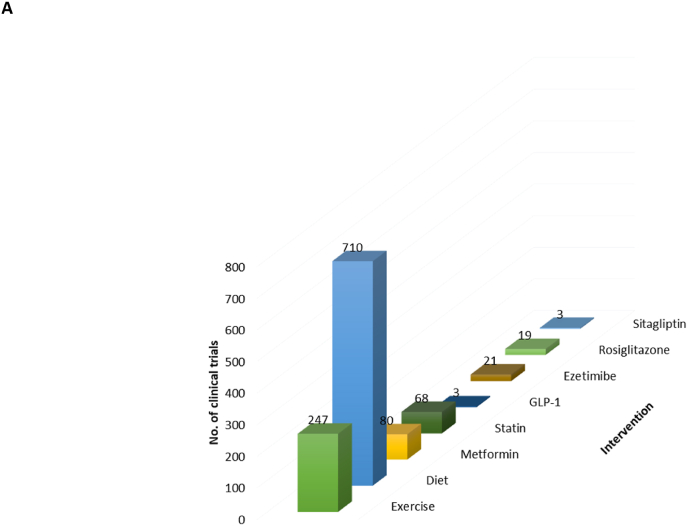

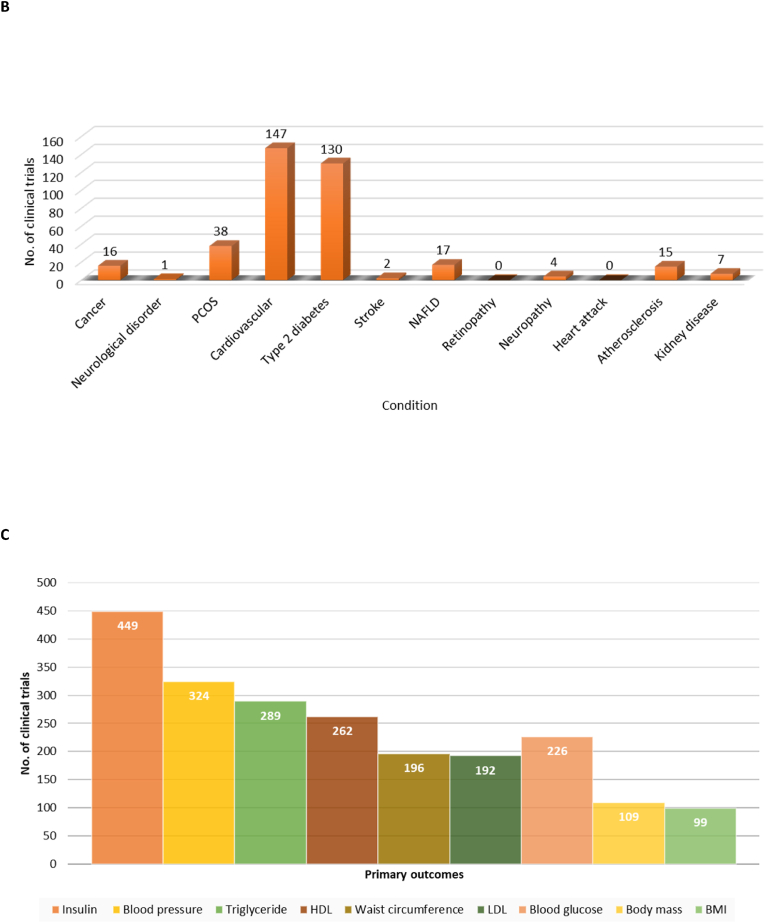
Diet and exercise were the predominant interventions being reported by clinical trials to address the prevalence of metabolic syndrome **(A)**. Moreover, metformin and statins were the leading pharmacological interventions in clinical trials looking at metabolic syndrome **(A)**. cardiovascular disease, type 2 (diabetes) and Polycystic ovary syndrome were the leading conditions reported by most clinical trials under the metabolic syndrome umbrella **(B)**. Blood lipid profiles, as well as insulins and glucose levels were the predominant diagnostic markers that were reported within diverse clinical trials of metabolic syndrome **(C)**.

Focusing on some of the known and promising metabolic syndrome interventions, analysis of the ICTRP data showed that diet and exercise with 710 and 247 studies were the prominent interventions being investigated, respectively (Fig. 7A). In terms of drug interventions, metformin was the leading drug intervention with 80 studies, followed by statins (68 studies), ezetimibe (28 studies), rosiglitazone (19 studies), GLP-1 agonists (3 studies), and sitagliptin (3 studies), respectively (Fig. 7A).

The metabolic syndrome is classified into diverse metabolic disorders [24], and from examining the 2379 trials registered, it was evident that most studies included CVDs, T2D, polycystic ovary syndrome, some cancers, non-alcoholic fatty liver disease, kidney disease, neuropathy, and neurological disorder (Fig. 7B). Among these studies, CVDs and T2D featured a higher number of research with 147 and 130 studies, respectfully (Fig. 7B).

Clinical trials have primary and secondary outcomes which are important to evaluate the effectiveness and safety of a new medical intervention or treatment. Since metabolic syndrome is a cluster of conditions, its diagnosis requires the occurrence of at least three or more conditions to be considered [25]. Fig. 7C showed that only few clinical trials focused on measuring the body mass (109 studies) and body mass index (BMI) (99 studies) when assessing as primary diagnostic features. Rather, a higher number of studies consider insulin (449 studies), blood pressure (324 studies), triglycerides (289 studies), and high-density lipoprotein (HDL) (262 studies) as prominent diagnostic markers (Fig. 7C). In trials investigating interventions for metabolic syndrome, it is crucial to evaluate these parameters.

## 4 Discussion

Growing research has shown that the metabolic syndrome is now a public and a clinical problem worldwide affecting the socio-economy statuses and causing considerable healthcare burden [23,26]. Despite the rising global prevalence of metabolic syndrome and its significant implications for public health, there is a need for more extensive and in-depth research to explore this research space. In fact, many aspects of metabolic syndrome, including its epidemiology, treatment strategies, and long-term outcomes, require further investigation and analysis. In our analysis of clinical trial data related to metabolic syndrome, several key findings have emerged. Initially, our dataset included 3236 studies, but after applying specific criteria, 857 studies were eliminated, leaving 2379 studies for further analysis. These trials have been registered since 1999, with a significant increase in registrations after 2004, aligning with the rising prevalence of metabolic syndrome. Notably, 2019 saw the highest number of registered studies at 176, reflecting the growing concern about this condition. Interestingly, this is in line with the growing trends in reported data showing alarming numbers of people with diverse metabolic complications, especially CVDs and diabetes, in different world regions [27,28].

Our analysis of global distribution revealed the United States as the leader in registered clinical trials, followed by Asian countries, notably Iran, China, and Japan. Conversely, certain Asian countries, including Bangladesh, Iraq, Lebanon, and Saudi Arabia, had minimal involvement in metabolic syndrome trials. Moreover, many regions in Central and Western Africa showed limited clinical trial engagement in this area, highlighting disparities in research activities. This shows discrepancy in terms of reliability of data emerging from these clinical trials, because most countries in Asia and Africa have seen a significant rise in cases related to the metabolic syndrome [29,30]. Regarding clinical trial registries, ClinicalTrials.gov dominated with 55.19 % of the studies, followed by the Iranian Registry of Clinical Trial (IRCT) and the Japan Primary Registries Network (JPRN). Registries like the Pan African Clinical Trial Registry (PACTR) and the Cuban Public Registry of Clinical Trials (RPCEC) had fewer registered trials. While gender and age distribution analysis revealed that most studies included both male and female participants, with a focus on adults. Although even gender and age distribution are expected [31], this is also another major discrepancy since clinical trials investigating or reporting on trends in metabolic syndrome in children were very limited, given the rising numbers in childhood obesity [32].

Further analysis of intervention models showed a predominance of parallel assignments, with treatment and prevention being the primary purposes being evaluated. Both diet and exercise interventions were prominently reported in these clinical trials, which is in line with research supporting the application of lifestyle modifications such as regular physical activity to reduce obesity and related complications [[33], [34], [35]]. In terms of therapeutic interventions, metformin and statins were the leading pharmacological drugs being reported within these clinical trials. This is consistent with the composition of diseases conditions, being mainly CVDs and T2D. These data verify the importance of both CVDs and T2D in contributing to the global burden of disease [36,37], while also highlighting metformin and statins as some of the leading therapies currently used to manage metabolic disease-related complications [[38], [39], [40]]. This hypothesis is consistent with clinical data indicating that primary diagnostic markers like insulin, blood pressure, triglycerides, and HDL, underpin the importance of using metformin and statins to manage complications of metabolic disease.

Nevertheless, beyond the use of metformin and statins, it is currently acknowledged that some of the recommendations for the treatment of individuals with the metabolic syndrome promote interventions aimed at reducing blood lipid profiles, including LDL cholesterol and triglycerides while increasing HDL cholesterol [[41], [42], [43]]. Nevertheless, research indicates that medications such as metformin, Glucagon-Like Peptide-1 agonists, and Dipeptidyl Peptidase IV inhibitors exhibit promise in diminishing the rate of advancement of conditions associated with metabolic syndrome [40,44]. In summary, our analysis provides insights into the landscape of clinical trials related to metabolic syndrome, highlighting regional disparities, dominant intervention models, and significant focus on CVDs and T2D.

The current study is not without limitations. Firstly, the predominance of data from ClinicalTrials.gov, which is based in the United States, may introduce bias and limit the global representativeness of the findings. Second, the study primarily focuses on adults, with limited attention to metabolic syndrome in children, despite its rising prevalence, potentially overlooking an important aspect of the condition. The study notes a focus on diagnostic markers like lipid profiles, insulin, and blood pressure in clinical trials, possibly neglecting other important outcomes such as body mass and body mass index. There is lack of extensive details about the research methodology employed, which may affect the assessment of the study's quality and validity. Most studies aimed to address general symptom relief while highlighting a need for additional well-designed treatment trials with rigorous methodologies in accordance with the World Health Organization's guidance for consistent evaluation and treatment [45]. Another limitation of this study is the presence of incomplete data in some of the registered clinical trials. Despite the benefits of transparency, previous studies have found the quality of information in registered clinical trials to be poor [46]. While the strengths are also acknowledged. The study provides a thorough examination of clinical trials related to metabolic syndrome, incorporating data from a wide range of sources and registries, offering a comprehensive overview of the field. It highlights the global significance of metabolic syndrome as a public health concern, emphasizing its association with CVDs and T2D, which is important for understanding its impact on a global scale. Importantly, by drawing upon data from 18 clinical registries, the study ensures a diverse and extensive dataset for analysis, enhancing the robustness of its findings. The study also identifies the most prominent interventions for metabolic syndrome, including diet, exercise, and pharmacological therapies like metformin and statins, offering valuable insights into current treatment approaches.

## 5 Conclusion

In conclusion, the current study highlights the critical importance of addressing the global issue of metabolic syndrome. This condition has emerged as a significant public health concern with far-reaching socio-economic implications and a substantial healthcare burden. Despite the increasing prevalence of metabolic syndrome worldwide, there is a clear need for more extensive and comprehensive research to bridge existing knowledge gaps. The analysis of clinical trial data reveals several key insights. In terms of intervention models, lifestyle modifications, including diet and exercise, feature prominently in clinical trials, highlighting their significance in managing obesity and related complications. Metformin and statins emerge as leading pharmacological therapies, reflecting the prevalence of CVD and T2D in the context of metabolic syndrome. However, there is growing recognition of other promising interventions, such as Glucagon-Like Peptide-1 agonists and Dipeptidyl Peptidase IV inhibitors, which offer potential in slowing the progression of metabolic syndrome-related conditions. These findings offer valuable information for researchers, policymakers, and healthcare professionals striving to address the challenges posed by metabolic syndrome on a global scale.

## Ethics approval and consent to participate

Not applicable.

## Consent for publication

Not applicable.

## Funding

The South African Medical Research Council supports this work on project code: 43500. The content hereof is the sole responsibility of the authors and does not necessarily represent the official views of the funders.

## Availability of data and materials

All data generated or analysed during this study are included in this published article.

## Not applicable.Patient and public involvement

Patients and/or the public were not involved in the design, or conduct, or reporting, or dissemination plans of this research.

## Ethics approval

Not applicable.

## CRediT authorship contribution statement

**Ndivhuwo Muvhulawa:** Formal analysis, Data curation, Conceptualization. **Phiwayinkosi V. Dludla:** Writing – review & editing, Writing – original draft, Formal analysis, Data curation, Conceptualization. **Musawenkosi Ndlovu:** Writing – review & editing, Formal analysis, Data curation. **Yonela Ntamo:** Writing – review & editing, Writing – original draft, Formal analysis. **Asanda Mayeye:** Writing – review & editing, Formal analysis, Data curation. **Nomahlubi Luphondo:** Writing – review & editing, Formal analysis, Data curation. **Nokulunga Hlengwa:** Writing – review & editing, Formal analysis, Data curation. **Albertus K. Basson:** Writing – review & editing, Formal analysis, Data curation. **Sihle E. Mabhida:** Writing – review & editing, Formal analysis, Data curation. **Sidney Hanser:** Writing – review & editing, Formal analysis, Data curation. **Sithandiwe E. Mazibuko-Mbeje:** Writing – review & editing, Formal analysis, Data curation. **Bongani B. Nkambule:** Writing – review & editing, Formal analysis, Data curation. **Duduzile Ndwandwe:** Writing – review & editing, Validation, Supervision, Resources, Formal analysis, Data curation.

## Declaration of competing interest

The authors declare that they have no known competing financial interests or personal relationships that could have appeared to influence the work reported in this paper.

## Data Availability

Data will be made available on request.

## Abbreviations

ANZCTR: Australian New Zealand Clinical Trials Registry
CVD: cardiovascular disease
HDL: high density lipoprotein
ICTRP: International Clinical Trials Registry Platform
IRCT: Iranian Registry of Clinical Trials
ISRCTN: International Standard Randomised Controlled Trial Number
JPRN: Japan Primary Registries Network
LDL: low density-lipoprotein
PACTR: Pan African Clinical Trials Registry
REPEC: The Peruvian Clinical Trials Registry
RPCEC: Cuban Public Registry of Clinical Trials
SLCTR: Sri Lanka Clinical Trials Registry
T2D: type 2 diabetes
WHO: World Health Organization
